# “Be my Voice” to break social stigma against domestic violence: The underestimated role of smartphone applications in protecting victims in developing countries

**DOI:** 10.3389/fpsyt.2022.954602

**Published:** 2022-08-04

**Authors:** Niloofar Saboury Yazdy, Ali Talaei, Mohammad Ebrahimi, Aida Ghofrani Ivari, Mohammad Amin Pouriran, Farhad Faridhosseini, Hossein Mohaddes Ardabili

**Affiliations:** ^1^Psychiatry and Behavioral Sciences Research Center, Mashhad University of Medical Sciences, Mashhad, Iran; ^2^Student Research Committee, Faculty of Medicine, Mashhad University of Medical Sciences, Mashhad, Iran

**Keywords:** smartphone, domestic violence, mental health, software, human rights, Iran, application, public health

## Introduction

Domestic violence (DV), which can be described as a pattern of assaultive and coercive behaviors, including physical, sexual, and psychological abuse committed within an intimate relationship, is a major global concern (1). DV-associated stigma can multiply the risk by preventing victims from help-seeking. Therefore, preventive measures should be taken in different stages. Here, after pointing out the significance of DV and its related stigma as a global social problem and briefly reviewing the available smartphone preventive solutions, we will shortly introduce “Be my Voice” as the first faculty-based Iranian app targeting DV victims.

DV can affect all genders and age groups, mostly women, children, the elderly, and other vulnerable individuals (2). It is estimated that overall, one in every three women experiences violence, physically and/or sexually, at some point in their lives (3). Studies have shown a higher prevalence of DV among women in Iran (66%) (4). This difference in prevalence may be rooted in social, economic, and cultural differences (5, 6).

DV can affect the victims not only physically but also mentally. These mental impacts can be severe and long-lasting. Post-traumatic stress disorder, depression, anxiety, substance use, and suicidal behaviors are examples of psychological problems caused by exposure to violence (7–9).

The COVID-19 pandemic has added even more complexity to this issue. Different studies have shown an increase in DV during COVID-19 confinement and lockdown periods (10–12). On the other hand, isolation at home has deprived many victims of telephone helplines, services, finance, informal social supports, or safe shelter, making the situation harder for the victims (10–12).

One of the problems facing this global issue is that we can only see a small proportion of DV while a significant amount of it remains unreported. Feeling ashamed and fear of being alienated by the society alongside unawareness, financial barriers, cultural beliefs, not feeling secure, and the threat of losing children and support leads to this under-reporting (13). Despite the high prevalence of DV throughout history, DV victims still experience high levels of stigma from different sources. Stigma is made of labeling, stereotyping, and separation that can cause status loss and discrimination. Different types of stigma faced by victims might include internalized stigma, anticipated stigma, enacted stigma, cultural stigma, and perpetrator stigma (14). Stigma in DV victims could cause negative feelings and shame due to isolation and loss of social status, therefore plays an important role in reducing help-seeking behavior (14).

Speaking of prevention, like other psychosocial issues, one must take four stages into consideration; primordial, primary, secondary, and tertiary prevention. Primordial prevention aims at risk factor reduction and typically gets promoted through laws and national policy (15). Primary prevention focuses on stopping conditions that support DV and encouraging conditions that inhibit DV. These measures might include promoting positive behaviors and skills to prevent DV through antiviolence campaigns, empowerment programs, and safety plan development (16, 17). Screening programs and referrals to legal services are examples of measures in the secondary prevention stage (17, 18). Long-term responses occurring after DV to deal with the lasting consequences of violence and offender treatment interventions are parts of tertiary prevention. Measures in this stage include providing mental and physical health interventions, safe-houses, and legal advocacies (17, 18). When measures are taken in all four stages together, they create a comprehensive response to DV.

## The role of smartphone apps in the prevention of domestic violence and related stigma

It is estimated that more than 6 billion people in the world own smartphones (19). The rapid advances in digital technologies and the worldwide dominance of smartphones and applications have created a great opportunity for delivering mental health services and interventions on a global scale (20). In recent years, especially during the COVID-19 pandemic, the role of technology-based interventions, including smartphones and applications, has become more prominent (21). Today more than 2.6 million apps for Android operating smartphones and more than 2.2 million apps for iOS devices are available (22). More than 40,000 of these applications are health-related (23). By only searching “domestic violence” in the Apple store, you can find more than 50 related apps. Table 1 shows a number of DV-related applications available in stores and their features. We searched the applications reviewed in two recent review studies in the Google play store and Apple store (24, 25). We then included the applications which were downloadable in any of the above stores in this table. At last, “Toranj,” the only DV-related application for Iranian women that we could find, was also added.

**Table 1:** Reviewing a number of DV-related applications and their features^*^.

**Applications name**	**Link**	**Country**	**Features**
Circle of 6	https://www.circleof6app.com/	USA	Sending a text message and GPS location to trusted friends. Direct access to information about sexuality, relationships, and safety. Direct access to national hotlines
Daisy	https://www.1800respect.org.au/daisy	Australia	Connecting people experiencing violence or abuse to supportive services in their local area. Safety features to help protect the privacy of people using it
myPlan	https://www.myplanapp.org/	USA	Safety planning and decision aid. Information about violence. Danger assessment tool
Bright Sky	https://www.hestia.org/brightsky	UK, available in 5 languages: English, Urdu, Punjabi, Polish, and Welsh	Providing information on support services. Risk assessment. Resources and information on domestic violence and abuse
Youth Pages	https://www.youthpagestoledo.org/	USA	Providing information about violence and available resources
RUSafe	https://wcspittsburgh.org/partner-violence/rusafe-app/	USA	Risk assessment. Connecting the user to domestic violence hotlines
Aspire News	https://www.whengeorgiasmiled.org/aspire-news-app/	USA	Sending messages or calling for help in crisis at the touch of a button. Resources for victims of domestic violence
Domestic Violence Prevention	https://www.applocker.navy.mil/#!/apps/29ACFD8A-D850-4BC4-AC0A-80E5898BE903	USA	Information about domestic violence and different ways to report it. Emergency contact to the hotlines
Positive Pathways	https://positivepathways.org.au/services/safety-and-wellbeing-app/	Australia	Voice recording features in crisis. Sending messages and GPS location to trusted friends. Connecting to the emergency services. Resources for victims of domestic violence
Gwen Alert	https://gwen.global/gwen-alert/	USA	Sending messages and GPS location to trusted friends
Toranj	https://www.toranjapp.com/en	developed in the USA for Iranian women	Providing emergency contact, legal and educational resources, free counseling centers, and relationship assessment
bSafe	https://www.getbsafe.com	USA	Sending GPS location, audio, and video to guardians. Voice and video recording. Producing fake calls

* *DV-related applications reviewed in two recent studies (24, 25), which were downloadable in Google play store and/or Apple store, were included in this table. Also, “Toranj,” the only DV-related application for Iranian women to our knowledge, was added to the table*.

In a recent study conducted by Moret et al. aimed to evaluate the prevalence and quality of free smartphone apps related to intimate partner violence and sexual violence prevention and response, 132 apps were evaluated and scored. Applications were categorized into eight groups based on intervention strategies: information/education, safety monitoring/tracking, goal setting/safety planning, location tracking, decision making, feedback, assessment, and others. The primary strategy of the majority of apps was information sharing and education, followed by safety monitoring and safety planning. The study stated that the included apps were of low to moderate quality (22).

In an article published in 2019, Laura Brignone and Jeffrey L. Edleson reviewed the smartphone applications related to the prevention of dating and DV. Thirty-eight applications were evaluated and rated based on a 27-point scale created by the author, assessing their performance as apps and as interventions for dating and DV. The ratings showed that four apps had low-quality, 17 had medium-quality, and 15 were classified as high-quality. This study also pointed out limitations of visibility and utility to prospective users. It was also suggested that applications developed by individuals with no connection to advocacy services or evidence-based practice could cause harm (24).

Probably one of the most reviewed applications related to DV is myPlan. This interactive decision aid and safety planning intervention app aims to assist feminine college students who have been violated in different ways. It also helps them by educating their family members and friends. This application allows the users to evaluate their relationships, consider their priorities, and develop a customized alternative plan. This app also offers the opportunity to connect directly to advocacy and mental health services (26, 27). A study showed that the use of myPlan significantly reduced users' experience of reproductive coercion and the risk of suicide compared to the control group, using usual safety planning. This study also showed a decrease in intimate partner violence over time in the myPlan group compared to controls (28).

HearMe is another application that aims to mitigate women's harassment. This app enables a tap-based emergency contact by sending a short message/phone call and generating an alarm sound in the destination device. It also provides information about nearby hospitals, police stations, and law assistance centers. Other features include audio recording, spy camera, and GPS location sharing (29).

SAP_MobAPP (sexual abuse prevention mobile application) is another application developed to educate primary school children in Korea about sexual abuse prevention. This app aims to educate the children to recognize child sexual abuse and empower them to prevent and protect themselves in such situations. This application provides users with animated scenarios and asks the user true/false questions. Evidence showed long-lasting improved awareness and skills to avoid child sexual abuse situations in children who used this application compared to the control group (30).

An interesting study in 2016 reviewed the responses of smartphone-based voice assistants to questions about mental health, interpersonal violence, and physical health. This study showed that only one out of four voice assistants reviewed recognized “I was raped” serious and connected the user to the hotlines. None of the four reviewed voice assistants recognized “I am being abused” or “I was beaten by my husband” as concerning statements (31). This problem seems to be resolved by the developers, and today all the three statements above are considered serious, and the user becomes connected to DV hotlines.

As mentioned earlier, victims of DV are prone to long-lasting psychological problems like depression and anxiety. Access limitations to mental health care and the shortage of mental health care staff in addition to the stigma toward medications and psychotherapies have limited their effectiveness (32–35). Smartphone-based interventions seem to be a promising alternative solution. A meta-analysis conducted by Firth et al. showed that smartphone apps had a significant effect on reducing depression symptoms compared to control groups (20). Another study showed that mobile apps for depression, besides their easy availability, have notable effects on patients suffering from moderate degrees of depression (36). It is reported that smartphone-based mental health interventions have the potential to be effective in generalized anxiety disorder (37, 38).

As discussed, technology-based interventions can be a promising solution to fight DV. Still it is also important to point out the role of technology as a tool for abuse. A study published in 2016 showed that offenders could abuse mobile technologies to stalk and harass women in the context of DV (39). Technologies like text messages, phone calls, GPS tracking, and social media enable the perpetrators to access and control the victims all day. Social media empowers offenders to abuse and humiliate victims, especially in sexualized ways (39). Another review study stated that ~50% of college students were either victims or perpetrators using communications technology in the context of an intimate partner relationship. It also stated that the development of digital technologies combined with intimate knowledge of the victim, permits the offender easy access to personal information alongside effortless abusive communication with the victims (40).

Taking all into account, technology-based interventions including smartphone applications play a dichotomous role in DV. Still, having in mind the abusive aspects of technology, we strongly believe that they are of great potential in combat against DV if used securely.

## “Be my Voice”: A tiny smartphone app aimed to prevent domestic violence, educate and support the victims

As mentioned earlier, the prevalence of DV has increased during the COVID-19 lockdown. On the other hand, the confinements have limited access to helplines and supporting facilities (10–12). Iran is no exception, and it seems that the prevalence and severity of DV in Iran have also increased, to the extent that three of the honor kills that happened in Iran during the first year of the lockdown were so drastic that went viral in the press for a long time (41–43).

In earlier paragraphs, we discussed the promising role of smartphone applications in raising awareness, fighting against DV, and providing support and help for victims. Traditions, beliefs, supporting systems, and even laws are deeply affected by sociocultural issues. Therefore, in order to make these applications efficient, they must be customized and culturally tailored for different countries. Considering the promising role of smartphone applications and also the lack of suitable and culturally tailored applications for Iranian victims, we decided to develop a smartphone app, named “Be my Voice” in Iran, a developing country with a high prevalence of DV and lack of supporting systems and NGOs (4). To our knowledge, this application is the first faculty-based DV app developed in Iran. This application is designed by medical students and psychiatry residents under the supervision of psychiatry professors at Mashhad university of medical science. This application offers the Iranian victims the chance to freely access information, plans, and supports compatible with local cultures and laws to fight the stigma surrounding DV.

**Figure 1:**
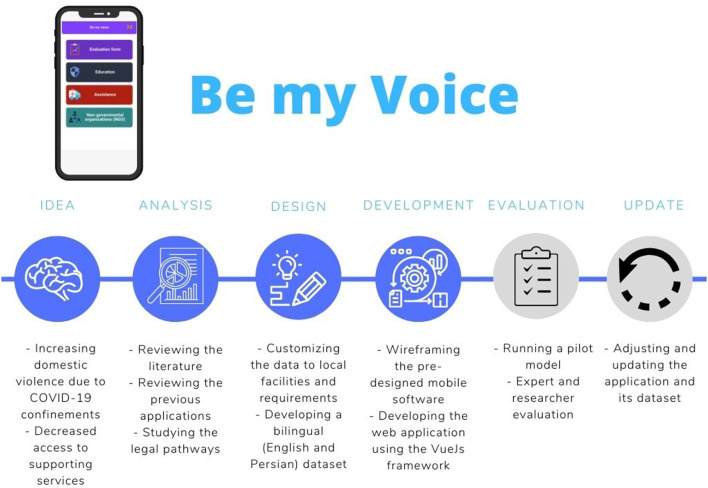
Different stages of developing Be my Voice application.

In order to design the app, we studied the legal pathways from which Iranian victims of DV may benefit. We also tried to create a list of different kinds of support and violence prevention instructions in distinct settings. Finally, after an extensive review of the literature and similar applications and adjusting our findings to local facilities and requirements, we wireframed the pre-designed mobile software. We then used the VueJs framework to develop a demo web application. Figure 1 shows different stages in developing Be my Voice application.

Reviewing the similar applications and adapting their useful features, we developed this bilingual easy-to-use app with multidimensional services for DV victims. A simple in-app questionnaire is provided to help victims discover their situation and whether they are victims of DV. This app also offers victims legal ways to claim their rights. Educatory materials on human rights, different types of DV, and supporting laws are provided in different categories for children and adults. This app also offers the victims the chance to make supporting networks and plans to leave home. In urgent situations, this app can connect the user to police, social emergency services, and pre-defined supporting persons. It also has the potential to provide a bridge between victims and supporting NGOs, which are highly lacking in Iran.

In order to provide security and privacy for the app users, we designed the application in a way that name and icon of the application and also the “help message” can be customized to keep it hidden and safe. The application is protected *via* a password and offers the user the chance to use it unidentified. It is also important to note that the user's information is kept on the servers anonymously.

Although the application is not yet fully developed and not in its final form we have made an online demo (accessible from: https://bemyvoice.netlify.app/) ready for hands-on in order to adjust and debug the application according to the experts' opinions before the first pilot study. It is also worth mentioning that in its first public presentation at the 38th annual congress of the Iranian psychiatric association, this app was selected for the 6th yearly Davidian Award for young psychiatrists^1^. The evidence provided by such apps can offer help and support for the government and in charge authorities to create efficient hotlines and supporting services. We are also planning to grab support from social charities and NGOs to provide clinical and legal services, especially for the victims with lower socioeconomic conditions.

## Conclusion

As discussed above, in order to prevent and manage the DV, measures must be taken in all four prevention stages. Technology-based interventions such as smartphone applications have the potential to provide free, available, and effective solutions in all those prevention stages. Excellent accessibility, affordability, availability, and anonymity are factors that make smartphone applications a promising solution for the prevention of DV and intimate partner violence. Especially in developing and underdeveloped countries, where the rate of DV is relatively higher (44), many people are unaware of their rights, and access to health care providers is limited.

The majority of the apps available in the Apple store and Google play store are in English and based in and customized for the USA or European countries, making them unsuitable for victims of DV in developing/underdeveloped countries. In order to increase the utility of these applications and make them more user-friendly, it is important that the applications are locally customized. Another critical aspect of developing health-related applications is the importance of supervision from healthcare-related specialists, which many applications lack. False information and unsupervised applications can cause more harm than benefit.

In this paper, we briefly reviewed the potential of smartphones in supporting DV victims and reducing its related stigma. We also introduced the first faculty-based Iranian DV prevention app in order to call for action and support from colleagues and organizations. We hope this will be a starting point for developing countries to join the club and utilize the extensive potential of DV management smartphone apps.

## Author contributions

NS has provided the main idea of the application. ME has designed and developed the application. MP has contributed in translating the dataset from Persian to English. AG has written the first draft and manuscript. HMA has contributed significantly to reviewing the manuscript critically. AT and FF have reviewed the manuscript. All authors contributed to the article and approved the submitted version.

## Conflict of interest

The authors declare that the research was conducted in the absence of any commercial or financial relationships that could be construed as a potential conflict of interest.

## Publisher's note

All claims expressed in this article are solely those of the authors and do not necessarily represent those of their affiliated organizations, or those of the publisher, the editors and the reviewers. Any product that may be evaluated in this article, or claim that may be made by its manufacturer, is not guaranteed or endorsed by the publisher.

## Footnotes

^1^ https://irpsychiatry.ir/fa/weblog/171082/بیانیه-هیات-داوران-دور-ششم-جایزه-استاد-داویدیان
